# Screening and Characterization of Thermostable Amylase-Producing Bacteria Isolated from Soil Samples of Afdera, Afar Region, and Molecular Detection of Amylase-Coding Gene

**DOI:** 10.1155/2021/5592885

**Published:** 2021-05-10

**Authors:** Semira Nureddin Yassin, Tamene Milkessa Jiru, Meera Indracanti

**Affiliations:** ^1^Department of Biotechnology, Institute of Biotechnology, University of Gondar, P.O. Box: 196, Gondar, Ethiopia; ^2^Department of Environmental and Industrial Biotechnology, Institute of Biotechnology, University of Gondar, P.O. Box: 196, Gondar, Ethiopia; ^3^Institute of Biotechnology, University of Gondar, P.O. Box: 196, Gondar, Ethiopia

## Abstract

Studying thermostable amylase-producing bacteria in extreme environments has a crucial role to overcome different industrial challenges. Afar Region is one of the hottest and salty areas, making it the home of extremophiles. This study aimed at screening and characterizing amylase-producing bacteria isolated from soil samples of Afdera, Afar Region, and detection of their amylase-coding genes. Thus, a total of 49 bacterial isolates were obtained from the collected soil samples. Out of these, three isolates (M2, M8, and M13) were selected on the basis of diameter of the average clear zone formation and time taken to decolorize iodine solution. Based on their morphological and biochemical characteristics, the isolates were identified as genus *Bacillus*. PCR amplification and detection of the amylase-coding gene confirmed the presence of the amylase gene in the three bacterial isolates. Optimum amylase production time for these isolates was 48 hrs (M13 and M8) and 72 hrs (M2) corresponding to the amylase activity of 0.67 U/mL for M13, 0.74 U/mL for M8, and 0.73 U/mL for M2 with an optimum temperature of 55°C. Studies on the effect of temperature revealed that the crude enzyme had a maximum activity and stability at 75°C, 70°C, and 65°C for isolates M13, M8, and M2, respectively. Additionally, amylase produced from all isolates retained more than 66.41% of their original activity after incubating them at a temperature range from 55 to 80°C for 50 min. Optimum pH for the activity of all crude amylases was in the range from 5 to 9 with a peak activity at pH 8. Their activity decreased significantly by the presence of Zn^+2^ and Mg^2+^; however, their activity increased by the presence of Ca^+2^. Moreover, the three crude amylases were stable (0–3 M) with NaCl concentration. Amylases of this finding with thermophilic and halophilic characteristics offer a wide range of applications in food, brewing, textile, starch, paper, and deterrent industries. Thus, identification of these *Bacillus* isolates at a molecular level and purification as well as detailed characterization of the types of amylases are recommended for effective utilization in different industries.

## 1. Introduction

Enzymes are biological catalysts, which initiate and speed up thousands of biochemical reactions in living cells. Enzymes are specific molecules due to the selective binding site each type of enzyme possesses for its respective substrate. They are applied in various fields such as in food manufacturing, fermentation, animal nutrition, detergent, cosmetics, brewing and textile to paper industries, pharmaceutical (medication), and as tools for research and development [1].

Among enzymes, amylase plays a significant catalytic role for the breakdown of starch into its monomeric compounds, the smallest being glucose [2, 3]. Although amylases can be produced biologically by plants, animals, humans, and microorganisms, enzymes derived from microorganisms are currently used in the majority of industries [4].

Microbial amylase is chosen over other kinds of amylase obtained from plants and animals due to its biochemical versatility, higher production rate, stability, and easy availability of a huge number of microbial strains [5]. Moreover, the ability to produce in bulk and ease at which it can be engineered to obtain enzymes are of desired characteristics [6].

Currently, amylase production has reached up to 30% of the enzyme market in the world and is continuously increasing [7]. Many microorganisms have been identified and chosen as a source of amylase production because of their availability, their rapid growth rates that lead to short fermentation cycles, their simplicity, their capacity to secrete proteins into an extracellular medium, the maximum yield they provide, and general handling safety [5, 8]. However, amylase extracted from bacteria and fungi has dominated various applications in biotechnology [3, 9], including *Bacillus* sp., *Lactobacillus* sp., *Proteus* sp., *Escherichia coli, Pseudomonas* sp., *Streptomyces* sp., *Aspergillus* sp. (e.g., *Aspergillus ficuum*), *Talaromyces emersonii*, and *Thermomyces lanuginosus* [10–12]. Thermostability is a desired feature of amylase to use for various industrial applications [2]. Thermophilic organisms are therefore of special interest as sources of novel thermostable enzymes [13]. Thermophilic bacteria (commonly many species of *Bacillus*) have been reported to be economically good sources of thermostable amylases [14].

Extremophiles are microorganisms that can grow and thrive in extreme environments, which are predominant conditions in a variety of industrial processes [15]. Many hyperthermophilic microorganisms possess starch-hydrolyzing enzymes in their genomes even though they live in environments where starch is rare [13]. The advantages of using thermostable amylases in industrial processes include the decreased risk of contamination and the increased diffusion rate [6]. The most common habitats for the thermophilic organisms are geothermally and volcanically heated hydrothermal systems, submarine saline hot vents, and hot springs soil and water [16]. It is believed that microorganisms found in these areas are capable of producing enzymes which can function at harsh (extreme) conditions [2, 10]. They are adapted at the molecular level to withstand these harsh conditions, and these biocatalysts are called extremozymes. Among these, thermostable amylases are one of the most important industrial enzymes [7].

The use of amylase is increasing from time to time globally including Ethiopia. Despite this, there is enormous interest in exploring enzymes with better properties such as thermostable raw starch degrading amylase suitable for industrial applications and cost-efficient production methods. In an industry that works at mild conditions, contamination and product batch-to-batch inconsistency is a major challenge which necessitates us to perform the fermentation activity at extreme conditions where the contamination is almost nil [11]. For this reason, most of starch-processing industries are designed to operate at elevated temperature, but the enzymes from microbes that survive only in mild environmental conditions easily get denatured in industrial conditions where the temperature, pH, and other parameters are higher or lower [15]. Research is still undergoing to find out the bacterial strain which produces more stable amylase enzyme with higher production rate within low cost. Therefore, the presence of starch-degrading enzymes that have appreciable stability at high temperatures is crucial. One of the important options is studying microbial populations that can live and reproduce in extreme environments.

Ethiopia is endowed with a range of natural geothermal sites and hot water springs. Afar Region is among the hottest regions in Ethiopia. Bacteria which can adapt and produce amylase can be present in this region. Hence, it is necessary to assess the potential of the geothermal site as a source of potential bacterial species for the production of active, heat-resistant, reusable amylase for the hydrolysis of starch in industries. Therefore, this study aimed at isolating and screening of thermostable *α*-amylase-producing bacteria and detection of their amylase-coding gene.

## 2. Materials and Methods

### 2.1. Description of the Study Area

For the current study, soil samples were collected from Afdera, Afar Region, Ethiopia. Afdera is one of the districts of Afar which is the hottest and driest place for most of the year with average temperature >27.5°C in monsoon to 45°C in the dry season. This district is included under the tropical zone with hot and desert climates. Its altitude ranges from 114 below sea level to 1300 meters above sea level. The annual rainfall varies from year to year, but it is mostly below 400 mm [17, 18].

### 2.2. Soil Collection and Isolation of Bacteria

Soil samples were collected randomly from Afdera, Ethiopia. From these soil samples, thermostable amylase-producing bacteria were isolated from soil by the serial dilution method [19]. In brief, one gram of the soil was dissolved in 9 mL of sterilized distilled water in different containers and given heat shock at 90°C for 15 min followed by cooling to room temperature. Then, suitable serial dilution (10^−1^–10^−6^) was undertaken [20]. A 0.1 mL sample was spread on nutrient agar plates followed by incubation at 50°C for 24–48 hrs until typical bacterial colonies were obtained. The colonies showing clear difference in morphology were further purified by subculturing and streaking on nutrient agar. The pure isolates obtained were picked and transferred into freshly prepared starch agar plates and incubated at 50°C for 48 hrs [21]. Then, they were stored at 4°C until required for further analysis and studies.

### 2.3. Screening and Selecting Amylase-Producing Bacteria

Purified thermophilic isolates that grew on starch agar were tested for amylase production [21]. Part of the colonies was subcultured in freshly prepared starch agar plates by the dot method and incubated at 50°C for 2 days. After incubation, the plates were flooded with 1% iodine solution (Gram's iodine: 250 mg iodine crystals added to 2.5 gm potassium iodide solution and 125 mL of water, stored at room temperature). The plates were then kept undisturbed for 5–10 min, and the iodine solution was discarded by decanting from each plate. Any formation of the clear zone around the colonies was observed, and the diameter was measured. The isolates which exhibited largest clear zones around them were selected [21]. The colonies were checked by inoculating them into test tubes having similar media, incubating at the same temperatures, and flooding with two drops of iodine solution, and the time taken to decolorize the iodine solution was recorded [22].

### 2.4. Identification and Characterization of Bacterial Isolates

#### 2.4.1. Macroscopic and Microscopic Characterization of Isolates

Isolates were further characterized by their morphology and biochemical properties according to Bergey's Manual of Determinative Bacteriology [23]. Colony morphology such as size, shape, color, and texture was determined. Gram staining, spore staining, and motility tests were performed following the protocols of Islam [4], Harley and Prescott [24], and Murray et al. [25], respectively.

#### 2.4.2. Biochemical Characterization

For characterization of bacterial isolates, various biochemical tests were carried out. Catalase and casein hydrolysis tests were performed following the method of Bennani et al. [26]. Citrate utilization, urea hydrolysis, and growth on anaerobic condition were also undertaken [24]. Oxidase and triple sugar iron tests were performed following the protocols of Tarrand and Gröschel [27] and Priyadarshini [28], respectively.

### 2.5. Amplification and Detection of Amylase-Coding Gene from the Bacterial Isolates

Amplification of the amylase-coding gene of the bacterial isolates was carried out further. Total genomic DNA was extracted using the GenElute™ Bacterial Genomic DNA Kit (Sigma-Aldrich, USA).

The bacterial isolates were first grown on Luria Bertani media for 24 hrs in an incubator. After incubation, 2 mL of the bacterial cell suspension grown was taken and centrifuged at 15,000 rpm for 10 min at 4°C temperature. Then, the resuspended cells were used to extract cellular DNA according to the protocol provided by GenElute™ Bacterial Genomic DNA Kit. Extracted DNA was stored at 4°C for further work.

#### 2.5.1. Measurement of DNA Concentration and Purity

DNA concentration was measured using a NanoDrop spectrophotometer. To the nanodrop, 1.5 *µ*L of nuclease-free water was used as the blank. The blank was removed by using a tissue paper, and 1.5 *µ*L of the sample was loaded [29]. DNA concentration was measured in ng/*µ*L. Agarose gel electrophoresis was also performed to qualitatively determine the purity of isolated DNA.

#### 2.5.2. Amplification of Extracted DNA Using Polymerase Chain Reaction

From extracted DNA, the amylase-coding gene was amplified using PCR (polymerase chain reaction). This was done using the following specific primer-forward: 5′AGTGCTGAA ACGGCGAACAAATCGAA3′and reverse: 5′CTCAATGGGGA AGA GAA CCGCTTAAG 3'. The primers were used by Prasad [29] with few modifications. The PCR mixture was prepared using Solis BioDyne 5x FIREPol^®^ Master Mix ready to load. 1 *µ*L of template DNA and 1 *µ*L of each primer were added right before loading the sample in the PCR machine. Reaction set up for PCR was carried out for 40 *µ*L reaction volume in a 0.2 mL thin-walled PCR tube.

The thermal cycle was programmed as follows: initial denaturation at 94°C for 2 min; 30 cycles of denaturation at 94°C for 30 sec, annealing at 50°C for 30 sec, extension at 72°C for 2 min, and a final extension at 72°C for 5 min. The PCR was carried out for 30 cycles. Afterwards, the amplicon was stored at −20°C for further work [30].

#### 2.5.3. Detection of DNA Using Agarose Gel Electrophoresis

After PCR, amplification was checked by horizontal electrophoresis in 1.0% agarose slab gel in Tris-Borate-EDTA (Ethylenediaminetetraacetic acid) or TBE buffer as the method used by Islam [2]. Agarose was dissolved in 1x Tris-Borate-EDTA buffer to give a final concentration of 1.0% agarose and was heated to dissolve in a microwave oven for about 30 sec. Then, it was allowed to cool down to about 50°C. In order to stain the DNA bands, 2 *µ*L of ethidium bromide stain was added to the cooled agarose and mixed. Agarose was then poured on to the tray previously set with the comb and allowed to solidify. A 5 *µ*L aliquot of the PCR product was loaded into the individual wells of the gel. A ladder of size 1 kb plus was used to ensure amplification of the desired gene and measure the exact product size. The DNA bands were photographed with the UV illuminator system, and the bacterial isolates were rated as starch degrading and nonstarch degrading based on whether there is amplification result.

### 2.6. Production of Amylase

Before the production of amylase, the inoculum was prepared by transferring a loopful of amylase-producing bacterial cultures in Erlenmeyer flasks (250 mL) containing amylase screening broth (starch broth) (50 mL) and incubated at 50°C in an incubator shaker at 250 rpm for 24 hrs.

Production of amylase was carried out using soluble starch as the substrate [31]. The selected isolates were separately cultured at 50°C temperature, pH 7, and 250 rpm for 48 hrs in 100 mL starch broth (1% starch, 0.5% peptone, and 1.5% yeast extract) after inoculated with 4 mL of an overnight bacterial culture. 6 mL of the sample was withdrawn from flasks after 48 and 72 hrs of incubation. The cell-free supernatant obtained after centrifuging the culture broth at 10,000 rpm for 10 min was used as the crude enzyme source. The crude extract was used for characterization of the enzyme activity and stability under different conditions.

### 2.7. Enzyme Assay and Characterization

#### 2.7.1. Amylase Activity Assay

Amylase activity was determined by measuring the release of reducing sugar from soluble starch. The reaction mixture contained 0.5 mL of the crude enzyme, and 4.5 mL of 0.1 M phosphate buffer (pH 6.0) was added to 2 mL of soluble starch (1%). After incubation at 30°C for 10 min in a shaking water bath, the reaction was stopped by the addition of 2 mL of 3,5-dinitrosalicylic acid [32]. The tubes were kept in boiling water for 15 min to develop color and then cooled to room temperature. The optical density (OD) of the resulting colored solution was measured at 540 nm against a blank. The enzyme assay in all cases was performed in triplicate, and the results are averages of the three determinations. A standard curve for maltose was prepared by the method performed to calculate the amount of maltose liberated. The OD readings were used to find out the unknown maltose concentration from the maltose standard curve. The amylase activity was measured. One unit of enzyme activity is defined as the amount of enzyme required to liberate one *μ*moL maltose per min under the assay conditions.

#### 2.7.2. Enzyme Production and Characterization

Enzyme production, activity, and stability were determined by characterizing substrate concentration (0.5%, 1.0%, 1.5%, 2.0%, 3.0%, and 4.0% starch solutions), temperature (40, 45, 50, 55, 60, 65, 70, and 75°C), incubation period (12, 24, 48, 72, 96, and 120 hrs), optimum pH using different buffers (acetate buffer, pH: 3 to 5; sodium potassium buffer, pH: 6 to 8, and Tris-hydrochloride buffer, pH: 9 to 11), NaCl concentration (0–8 M NaCl), and different metal ion concentrations (1.0, 5, and 10 mM of salts of CaCl_2_, MgCl_2_, and ZnCl_2_). Also, the crude enzyme stability was measured by incubating crude amylase (10–60 min) at various reaction temperatures (55–105°C). Enzyme production, activity, and stability were determined after growing the thermostable bacterial isolates on starch broth (1% starch, 0.5% peptone, and 1.5% yeast extract) except the test for substrate concentration. One parameter was tested at a time. Residual amylase activity was measured under assay conditions. Residual amylase activity was calculated using the following equation:

(1) $$\begin{array}{c} \text{Residual activity}=\frac{\text{Individual enzyme activity at each concentration}}{\text{The highest enzyme activity in the block}}\times 100\%. \end{array}$$

The highest enzyme activity in the block.

#### 2.7.3. Data Analysis

The enzymatic activity assay was performed in triplicate with three independent replicates, and data presented in figures and tables are the average of three parallel experiments. Statistical evaluation for significant differences between mean values was performed using one-way ANOVA at the 95% level (*P* ≤ 0.05) with the help of Statistical Package for Social Sciences (version 22).

## 3. Results

### 3.1. Isolation and Screening of Amylase-Producing Bacteria from Soil

In this study, soil samples for the isolation of bacteria were collected from hot temperature area, Afdera, Ethiopia. Based on colony morphology, a total of 49 distinct colonies were isolated. From these, using the iodine solution, 15 (31%) isolates were observed to give a zone of clearance around their colonies. As it can be seen from Table 1, the isolates showed a great variation in the size of the clear zone of hydrolysis they produce on starch agar plates ranging from the least 1.1 mm to the largest 5.9 mm.

### 3.2. Selection of the Best Amylase-Producing Bacteria

From the total of 15 positive isolates, three isolates were selected for further investigation. The selection of potent bacteria was done by comparing the isolates with each other in terms of both their diameter of the clear zone of hydrolysis and time to decolorize the iodine solution (Table 1). The results showed that the isolates with a higher clear zone of hydrolysis also gave higher amylase activities. This step resulted in the selection of three potent isolates.

**Figure 1:**
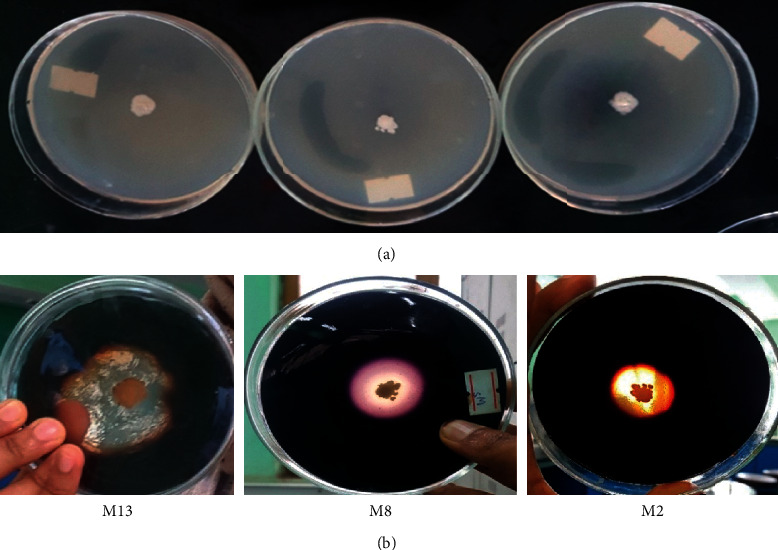
Zone of hydrolysis of starch by the three selected isolates before (a) and after (b) the addition of iodine solution.

The three isolates, namely, M13, M8, and M2, produced the largest ratio of halo diameter (5.96 ± 0.057, 4.96 ± 0.057, and 4.73 ± 0.115 mm) and shorter time to decolorize the iodine solution (6.67 ± 0.577, 9.67 ± 0.577, and 12.33 ± 0.577 sec) (Figure 1), respectively, than the other screened isolates and were selected for further study. Table 1 demonstrates the results obtained from the starch agar medium and the comparison of average clear zone ratio of amylase-producing bacteria on starch agar plates. The diameter of average clear zone ratio and time to decolorize iodine solution which was produced within different isolates showed significant difference (*P* ≤ 0.05).

### 3.3. Identification and Characterization of the Selected Bacterial Isolates

**Figure 2:**
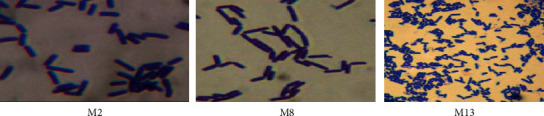
Results of Gram's staining for the selected isolates.

**Figure 3:**
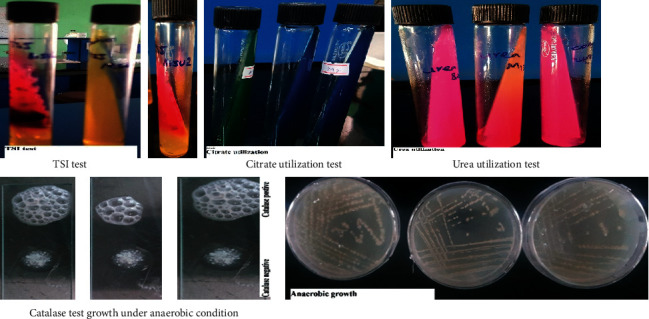
Results of some of the biochemical tests performed.

The morphological and biochemical characteristics of the bacterial isolates are summarized as follows (Tables 2 and 3). Figures 2 and 3 also show results of Gram's staining and some of the biochemical tests performed against the three isolates. According to the presumptive identification results, it was assumed that isolates M2, M8, and M13 might belong to the genus “*Bacillus.*”

### 3.4. Amplification and Detection of Amylase-Coding Gene

#### 3.4.1. Measurement of DNA Concentration and Purity

**Figure 4:**
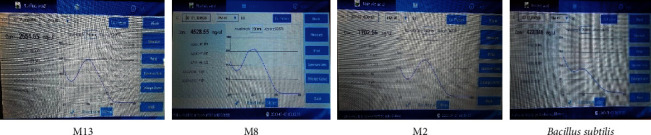
Quantification of DNA using nanodrop.

**Figure 5:**
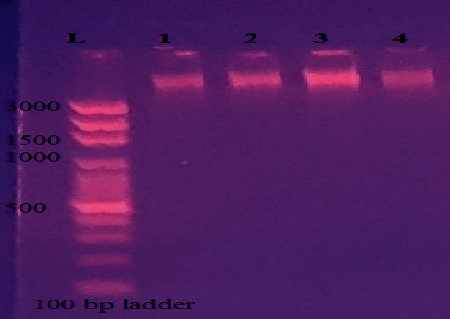
DNA bands visualized on agarose gel electrophoresis.

DNA concentration and purity were checked by nanodrop (Figure 4) and agarose gel electrophoresis (Figure 5). The quantification (concentration of DNA) result for the selected isolates was 2654.56, 4528.55, 1702.56, and 422.346 ng/*µ*L with a quality of 1.9, 1.91, 1.86, and 1.9 at A_260_/A_280_ ratio for M2, M8, M13, and *Bacillus subtilis* (positive control), respectively (Figure 4).

Qualitative estimation of the isolated DNA was done by running on agarose gel to determine the presence of pure DNA using agarose gel electrophoresis. It can be seen that the genomic DNA bands of isolates M2, M8, M13, and *Bacillus subtilis* (Figure 5) migrated along the lanes of wells 1, 2, 3, and 4, respectivly. “L” indicates DNA ladder.

#### 3.4.2. Amplification and Detection of Amylase-Coding Gene

**Figure 6:**
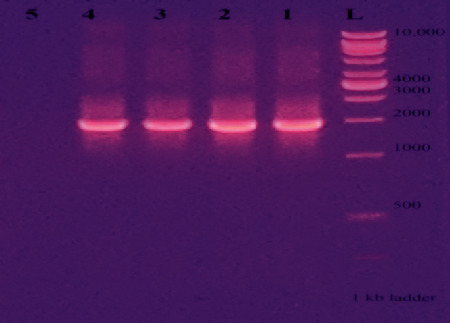
Visible amylase-coding gene bands over the UV transilluminator on 1% agarose gel electrophoresis.

It can be seen that the amylase-coding gene bands of isolates M13, M8, and M2 (Figure 6) which migrated along the lanes of wells 2, 3, and 4, respectively, are slightly lower than 2000 base pairs (bp) or 1855 bp when compared to the ladder which migrated along the lane of well “L.” Lane 1 indicates the DNA band of the positive control (*Bacillus subtilis*), and lane 5 shows that no band can be seen for the negative control.

### 3.5. Amylase Production

#### 3.5.1. Effect of the Time Course on Amylase Production

**Figure 7:**
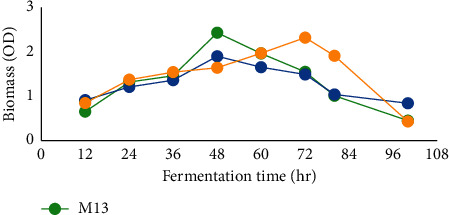
Cell growth with respect to different incubation periods.

The effect of the time course on cell growth was detected after 12 hrs of incubation. Except M2, in the rest two isolates (M13 and M8), maximum cell growth (optimum time course) was observed at 48 hrs of incubation, but in M2, maximum cell growth was observed at 72 hrs of incubation (Figure 7). In all isolates, extensions of the time course beyond the optimum time course have resulted in the decrease of cell biomass. Among studied isolates, there was significant (*P* ≤ 0.05) difference in biomass within different fermentation hours.

**Figure 8:**
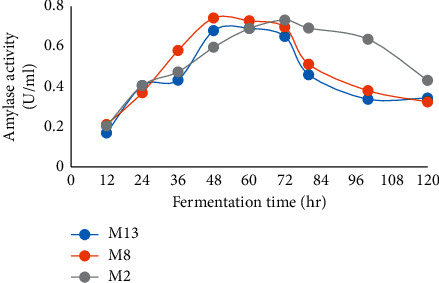
Effects of the time course on amylase production from selected bacterial isolates.

The effects of the time course on amylase production were observed after 12 hrs of incubation, the optimum duration period for the maximum amylase production from the three isolates was observed at 48–72 hrs, whereas the maximum amylase production from isolate M2 was observed between 72 and 96 hrs (Figure 8). There was a reduction in amylase activity of all isolates after 72 hrs which is expected as the microbial biomass started to drop after 72 hrs (Figures 7 and 8). The amylase activity produced from different isolates within different incubation periods showed significant difference (*P* ≤ 0.05).

#### 3.5.2. Effect of Cultivation Temperature on Amylase Production

**Figure 9:**
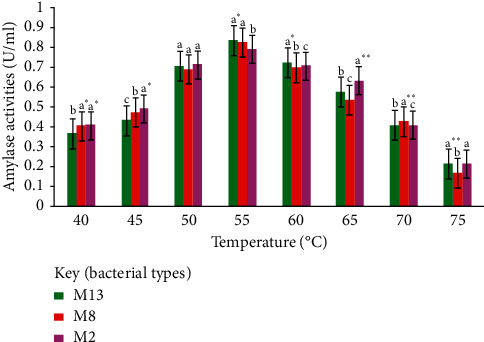
Effect of incubation temperature on the amylase production of M13, M8, and M2; mean values with the same letter are not significant (*P* ≥ 0.05); ^*∗∗*^significant at *P* ≤ 0.05.

The optimum temperature for the production of amylase by isolates M13, M8, and M2 was found to be 55°C, which resulted in amylase activities of 0.84 U/mL, 0.82 U/mL, and 0.79 U/mL, respectively (Figure 9), beyond which the enzyme activity was reduced gradually. The amylase activity produced from different isolates within the same temperature did not show significant difference (*P* ≥ 0.05).

#### 3.5.3. Enzyme Characterization

**Figure 10:**
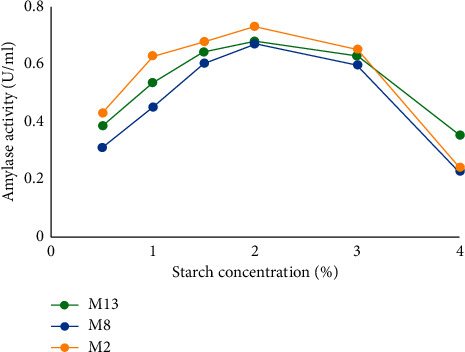
Effect of starch concentration on amylase activity.

**Figure 11:**
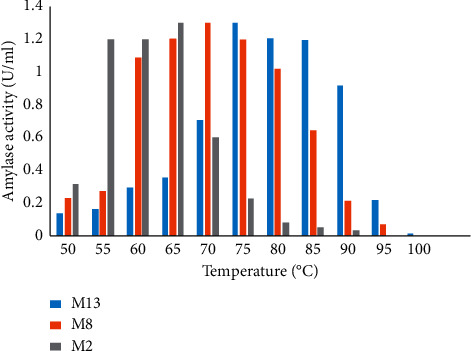
Effect of temperature on amylase activity of M13, M8, and M2.

(i) *Effect of Substrate Concentrations on Amylase Activity*. As shown in Figure 10, amylase activity increased with the increment of starch concentration from 0.5 to 4%, and it declined after this. In this study, the highest amylase activity was observed at 2% starch concentration. There was significant difference (*P* ≤ 0.05) in the amylase activity in different starch concentrations.

(ii) *Effect of Temperature on Enzyme Activity and Stability*. The effect of temperature on the activity of amylase was studied by incubating the culture filtrate with the substrate at temperatures ranging from 50 to 100°C. The highest amylase activity for isolates M13 and M8 was recorded at 75 and 70°C, respectively, whereas for M2, it was 65°C at 10 min incubation time (Figure 11). The amylase activity within the enzyme of the same isolates at different temperatures showed significant difference (*P* ≤ 0.05).

**Figure 12:**
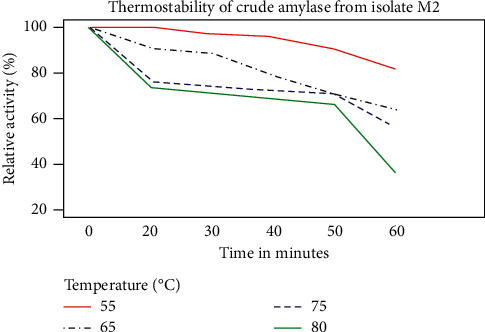
Thermostability of crude amylase from M2. Results are the average of the triplicate experiment.

**Figure 13:**
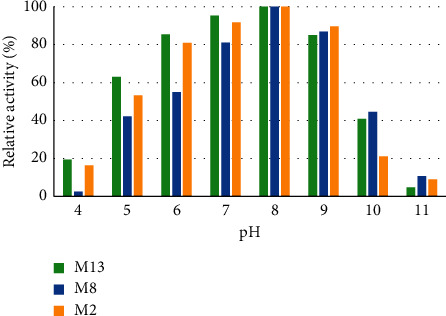
Effect of pH on amylase activity of the selected isolates.

**Figure 14:**
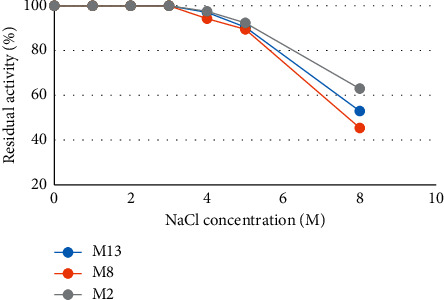
Effect of NaCl concentration on the stability of crude amylase.

The crude enzymes were characterized at different temperatures (55–105°C) for 10–60 min of incubation at each temperature. Amylase produced from all isolates retained more than 66.41% of their original activity after incubating at a temperature range from 55 to 80°C for 50 min. Furthermore, amylase produced from isolate M13 retained 80.94% of its original activity after incubating at a temperature of 80°C for 60 min. Further incubation of amylase produced from M13, M8, and M2 at 75°C for 50 min has retained their original activity by 85.94%, 76.49%, and 70.54%, respectively (Figure 12). The result also indicates that crude amylase of the three isolates loses all of their original activity after 20 min of incubation period above 100°C. The amylase stability of the enzyme produced from different isolates within different incubation periods of temperature showed significant difference (*P* ≤ 0.05).

(iii) *Effect of pH on Enzyme Activity*. The effect of pH on the activity of amylase was studied by incubating the culture at pH values ranging from 4 to 11. With an increase in pH, the activity of the enzymes was observed to increase as well. Optimum pH for the activity of the three isolates was in the range of 5–9 with a peak activity at pH 8. However, the enzyme activity decreased after pH 9. Amylase from isolates M13, M8, and M2 retained 85, 86.85, and 89.59% of their activity at pH 9, respectively (Figure 13). The amylase activity of enzymes from different isolates showed a significant difference (*P* ≤ 0.05) under different pH values

(iv) *Effect of NaCl Concentration on the Stability of Amylase*. The effect of salt on enzyme activity is presented in Figure 14. Salt tolerance of each enzyme was evaluated by incubating each enzyme in the presence of different NaCl concentrations (0 to 8 M NaCl). Except isolate M13, the rest two isolates' residual activity was 100% starting from 0 to 3 M NaCl solution, but isolate M13 retained 99.98% of its original activity at 3 M NaCl concentration. The residual activity of all isolates sharply decreased after 4 M NaCl concentration, i.e., 52.96, 45.44, and 62.96% for M13, M8, and M2, respectively, at 8 M NaCl concentration.

(v) *Effect of Metal Ions*. The effect of additional metal ions on amylase activity was determined in the presence of various metal ions at a concentration of 1 mM, 5 mM, and 10 mM (Table 4). Among the divalent cations tested, the activities of amylases from the three isolates decreased significantly by all of the three concentrations (1 mM, 5 mM, and 10 mM) of Zn^2+^ and Mg^2+^, but the activity of an enzyme in all isolates was higher in the presence of 5 mM (M8 and M2) and 10 mM (M13) Ca^2+^. 10 mM Mg^2+^ inhibited 23.1%, 21.7%, and 22.4% of amylase activity of M13, M8, and M2, respectively. The amylase activity of the enzyme produced from different isolates within different metal divalent cations' incubation showed significant difference (*P* ≤ 0.05).

## 4. Discussion

Globally, amylase is frequently used in food, textile, detergent, and paper industries. In addition, pharmaceutical and chemical industries use amylase on a regular basis to yield their products [6]. Enzymes' production from thermophilic hosts has several advantages compared to mesophilic hosts, including lower contamination risk, increased substrate and product solubility, and temperature optima of these bacteria matching those of enzymes used for simultaneous saccharification and fermentation [33]. Due to having this property, the proteins or enzymes from such organisms generally show thermostability/activity at high temperatures [34]. For this reason, thermophilic amylase-producing bacteria are needed. At present, enzyme-based hydrolysis using thermostable amylases is widely used for starch liquefaction [35]. Since industrial amylase is usually extracted from bacteria and fungi, it is mandatory to isolate a local high amylase-producing strain. Amylase-producing microorganisms can be isolated from different habitats, but soil is known to be the best source of amylase-producing microorganisms. Due to the availability of various types of bacteria in soil, it was chosen as the source of bacterial isolation and is sometimes considered as the storehouse of amylolytic microorganisms [36]. The soil collected for this study was from Afdera, Afar Region, Ethiopia. In this study, out of all isolates, 15 were amylase producers. Based on their starch-degrading efficiency and a short time to decolorize iodine in starch broth, only three isolates were selected and designed as M2, M8, and M13. They were determined by starch hydrolysis test and detecting any clear zone production around the colonies by adding iodine solution. The clear zones produced were due to the absence of starch which was hydrolyzed by the amylase enzyme excreted by the bacteria. This indicates the possible capability of the isolates to produce potential amylase, which could be used for different industrial applications. In addition, this result implied the soil of the study area is a rich source of thermophilic bacteria that could produce amylase and other important industrial enzymes.

The next step of the study was identification of the bacterial species by morphological and biochemical characterization. This was done for confirmation of the genus to which the isolates belong to. Bacterial cells were observed under a light microscope after both Gram's staining and endospore staining. Based on Bergey's manual of determinative bacteriology, the isolates M2, M8, and M13 were classified as Gram-positive, rod-shaped arranged in chains, and spore-forming bacterial species that may belong to the genus *Bacillus* [23]. It is estimated that *Bacillus* sp. enzymes comprised about 50% of the total global enzyme market [37]. Although members of the genus *Clostridium* are also spore-forming bacteria, the three isolates of this study do not belong to this genus as they are catalase positive and capable of growing under both aerobic and anaerobic conditions. Also, these isolates have been identified as motile. Various biochemical tests were performed to verify the biochemical characteristics of the bacterial isolates through which it was determined that apart from amylase, these bacterial isolates can also produce the enzymes catalase, urease, and oxidase (except M8).

These were then processed for DNA isolation and amplification of amylase-coding gene by PCR. DNA extraction method suitability is determined by characterizing the extracted DNA's quantity and quality. DNA quantity is a sign of extraction efficiency, and quality parameters (purity and intactness) indicate DNA is free of PCR inhibitors. DNA was isolated from the bacterial isolates which showed maximum amylase production. Quantification of DNA was carried out by nanodrop to determine the purity of DNA. This result indicated with the purity ratios within the accepted range of 1.8–2.0 for A_260_/A_280_ ratio. A similar result was reported by Olson and Morrow [38]. A_260_/A_280_ ratio is the better indicator for PCR, and ratios between 1.8 and 2.0 for A_260_/A_280_ are accepted as indicating pure DNA [39]. The concentration of DNA (in ng/*µ*L) for each sample was also determined from the NanoDrop spectrophotometer and gave good concentration for PCR. DNA shearing was evaluated using agarose gel electrophoresis. The qualitative estimation of DNA on 1% agarose gel gave single, sharp, and distinct bands devoid of any smear. Thus, DNA of good quality without any degradation was successfully isolated from all selected isolates.

Specific primers, namely, AmyF and AmyR, were used for the amplification of the amylase gene in the DNA sequence of the isolated bacterial species. These primers are designed to have base sequences complimentary to the sequences of the amylase-coding gene and hence attach to the area where the gene is located to perform replication. The same primers were used with some modifications in the study conducted by Prasad [29] for the detection of the amylase-coding gene. The amplification was performed using the PCR method. Afterwards, the bands of amplified gene sequences were visible after the PCR products were run on 1% agarose gel and viewed on the UV transilluminator. As expected, the acquired amylase gene DNA band from the three isolates was observed to have a size of less than 2000 bp (1855 bp) (equal size with the positive control (*Bacillus subtilis*) amylase-coding gene). The microbial production of amylase is beneficial as it is economical, gives high yield, and can be engineered to produce enzymes with desired characteristics. The gene for amylase production might further be cloned into other organisms, and the process of amylase production can be optimized.

The production of amylase from microorganisms under submerged fermentation is greatly affected by numerous physicochemical parameters [40]. The rate at which starch is broken down by amylase depends on various parameters. Characterizing an enzyme leads to the determination of optimum fermentation conditions for that enzyme. Furthermore, the inhibitory concentrations of metal ions and salts for that particular enzyme can be checked. The properties of amylase should meet its application, and hence, it is mandatory to check its optimum conditions which can be done via characterization [4].

The time course for reaching the highest enzyme level is associated with the characteristics and growth rate of the selected isolates. The optimum time for amylase production from the three isolates was found to be 48 hrs for M13 and M8. Similar findings were also observed on *Bacillus subtilis* and *Bacillus* sp. DLB9 [41, 42]. By contrast, 72 hrs of growth time was the optimum time for isolate M2 for amylase production and then declined. This might be due to the decrease in microbial growth associated with the depletion of available nutrient, production of toxic metabolites, and autolysis caused by amylase produced as reported by other studies, Haq et al. [43]. Different incubation periods have been reported by other studies for maximum amylase production: 24 hrs in *Bacillus cereus* [44], 48 hrs in *Bacillus subtilis* JS2004 [45], 72 hrs in *Bacillus subtilis* NCTC10400, *Bacillus cereus* ATCC14579, and *Bacillus licheniformis* [37, 46], and 120 hrs in *Bacillus* sp. [47]. Findings of the corresponding experiments revealed that the time course of enzyme production varied with the source of isolation, type of the strain, genetic makeup of the strain, and cultivation condition. Determination of the period of bacterial growth and amylase productivity are significant to optimize the time of product recovery as well as beneficial in managing production costs associated with incubation time.

Temperature had profound effect on amylase production. The isolates have been grown and revealed high amylase production in the temperature range of 50–60°C with maximum amylase production at 55°C. This result was similar to the results reported by Mrudula and Kokila [44] from *Bacillus* species, but amylase reported by Khusro et al. [48] and Gebreselema [42] demonstrated that maximum amylase production for *Bacillus* sp. from the poultry source and soil, respectively, ranged from 35 to 40°C. The variations among our reports and the previous studies might be due to different sources of bacterial isolation as well as types of strain. Our result shows the thermophilic nature of the isolated bacteria which is an indication of the potential applicability of the selected isolates to produce thermostable amylase that might work at high temperature in different starch-processing industries.

The effects of substrate concentration were studied as a result of this, highest amylase activity was observed at 2% starch concentration. This result was in agreement with that of Alli et al. [49], Oboh [50], and Sahoo et al. [51]. The starch concentration beyond 4% resulted in declined amylase activity. This might be due to metabolizing capacity of the isolates within the short period of time when the starch concentration was increased. The present findings are in corroboration with the report of earlier findings on amylase activity obtained from *Bacillus* species [52]. According to this finding, the concentration of starch may not be surpassing over 4%. Studies have also indicated that a high carbohydrate concentration repressed enzyme production. For this reason, carbohydrates can be added either continuously or in aliquots (fed batch) throughout the fermentation to supplement the exhausted component [53]. These data are significant to optimize the fermentation process within this range of substrate concentration.

The presence of active amylase in a wide range of temperatures is one of the essential features that makes amylase useful for various industrial applications. In this study, the crude enzymes were found to be active in a wide range of temperatures. Even though the enzymes were active in all the temperatures, M2 was more active at temperatures between 55 and 65°C with a peak at 65°C, while M8 was active at temperatures between 65 and 75°C with a peak at 70°C. The present study report was in complete agreement with the finding of Yasser et al. [54] in which the maximum activity of amylase produced by *Bacillus* species and *Bacillus subtilis* was achieved at 65 and 70°C, respectively, but the optimum amylase activity observed in the two isolates was lower than that of amylase isolated from *Bacillus* species, which was at 75 and 95°C [11, 22]. Moreover, isolate M13 was active between 75 and 85°C with a sharp decline above 85°C with a high activity at 75°C; it was high temperature as compared to the active temperature for the two isolates (M8 and M2). A similar result was also reported for *Bacillus licheniformis* isolated from cassava steep water [55]. However, the optimum temperature of amylases reported in this study was lower in comparison with amylases reported by Aynadis et al. [22] from the species of *Bacillus*. Moreover, the enzyme produced from those isolates revealed high thermostability, which was more than 66.41% of their original activity after incubating at a wide range of temperatures (55–80°C) for 50 min. This potential enzyme activity, particularly at 80 C, implies its usefulness in various industrial applications. Furthermore, amylase produced from isolate M13 retained 80.94% of its activity after incubating at a temperature of 80°C for 60 min. This isolate was more stable than amylase produced from M8 and M2. Further incubation of amylase produced from M13, M8, and M2 at 75°C for 50 min retained their activity by 85.94%, 76.49%, and 70.54%, respectively. These enzymes were less stable than alpha-amylases produced from *Bacillus* species, which retained more than 90% of the original activity after incubating at 90°C as reported by Saad [56]. Besides, the enzymes lost their original activity after incubating them above 100°C for 20 min. This might be because above their optimum temperature, the enzymes' structure begins to break down or disrupt the shape of the active site, which will reduce its activity or prevent from working. Thus, to use the enzymes with their appreciable stability for various applications, it is important to consider suitable temperature for the enzyme.

pH is another factor that markedly affects the activity of amylases [57] and has significant implication on the application of enzymes. When the enzyme was characterized to identify optimum pH, it was observed that amylase from the three isolates was active from 5 to 9. The highest activity was shown at pH 8. The soil itself in Afar Region is mainly alkaline, and this may be the reason why amylase from these isolates works best at alkaline pH. This suggests the enzymes would be useful in the process that requires a wide pH range from slightly acidic to alkaline medium. The study reported in this finding is more or less similar to the investigation report of Aynadis et al. [22] and Mrudula et al. [58]. On the contrary, Dipali and Ajit [3] demonstrated that the bacterial strain *Bacillus* sp. WA21 optimum pH of amylase obtained was 6 which is less than that found in this study. A higher optimum pH value of amylase was obtained within the range of pH 9 to 10 as reported by Simair et al. [16] who worked on the *Bacillus* strain, GM890. Moreover, amylase from isolates M13, M8, and M2 also retained 85, 86.85, and 89.59% of their original activity at pH 9, respectively. This result is in line with Sahoo et al. [51] who worked on *Bacillus* sp. MRS6, isolated from municipal solid waste. Also, all enzymes of the isolates revealed relatively better activity at pH 10 than pH 4. This indicates that the enzyme could work at alkaline conditions, which has huge potential for the detergent industry. It is known that enzymes in detergents have potential abilities to remove tough stains without any environmental effects [59]. Currently, such type of enzyme formulations is widely used for laundry and automatic dishwashing in order to remove starchy food substances derived from gravies, potatoes, chocolates, custard, and other smaller oligosaccharides [60].

Salt tolerance of crude amylases in this study indicated that the addition of 0–3 M concentration of NaCl did not affect the activity of the three crude amylases. However, when NaCl concentration was increased beyond 3 M, the activity decreased, but retained above 89.45% till 5 M salt concentration. This clearly indicates that the enzyme is halotolerant and is comparable with a few halophilic amylases reported in the literature [51]. Halotolerant bacterium *Chromohalobacter* sp. TVSP 101 [61] *α*-amylase was shown to exhibit 90% of the relative activity till 2.5 M. In addition, Kiran and Chandra [62] reported *Bacillus* sp. strain TSCVKK which retained 100% activity at 1.7 M NaCl. However, Berhanu [47] observed the highest halotolerant amylase-producing *Bacillus* species which were active at 0–5 M NaCl concentration. Therefore, this halophilic stability and adaptation of halothermophilic strains with capability to produce amylase suggested that these strains could be good choices for some biotechnological applications.

External factors such as cations and additives have been known to affect the activity of the enzyme. Some trace elements are needed by different microorganisms for their growth as well as for many enzyme-catalyzed reactions [22]. Commercial thermostable amylases currently used by the starch industry require Ca^2+^ for activity and stability, may act as a cofactor [63]. Therefore, Ca^2+^ is normally added to the reaction mixture to stabilize the enzyme. This report supports our result. In our study, in the presence of Ca^2+^, the percent residual activity was greater than 100% in the three enzymes. This indicates that the addition of 5 mM and 10 mM concentrations of Ca^2+^ slightly activated enzyme catalysis. Crude amylase from M13 was more activated during the addition of 10 Mm Ca^2+^; rather, the enzymes produced by M8 and M2 only needed 5 mM Ca^2+^. Similar results were reported by Aynadis et al. [22]. However, our reports were against the observation of Atsbha et al. [11], who demonstrated that activities of both enzymes from two *Bacillus* species in Afar, Ethiopia, were decreased in the presence of Ca^2+^, Zn^2+^, and Mg^2+^ ions in all concentrations (1, 5, and 10 mM). Both Zn^2+^ and Mg^2+^, by all of the three concentrations (1 mM, 5 mM, and10 mM), significantly decreased the activity of the crude enzymes. Most *α*-amylases from different sources are inhibited by metal cations such as Mg^2+^, Mn^2+^, Cu^2+^, and Zn^2+^ which is matching with our results. Similar reports were also observed by Aynadis et al. [22] and Atsbha et al. [11]. In addition, Yaseer et al. [54] reported that Zn^2+^ and Cu^2+^ had inhibitory effect on amylase activity. Ahmad et al. [64] also reported that the enzyme activity of *α*-amylase from *Bacillus* species was inhibited by Zn^+2^ and EDTA.

## 5. Conclusion

The overall finding of the present study shows that it is possible to isolate potential thermophilic amylase-producing bacteria from the soil of Afdera, Afar Region. All of the isolates were Gram-positive, spore-forming rods. In addition, by using morphological and biochemical characteristics, they might belong to *Bacillus* sp. *Bacillus* sp. are known as potent producers of amylase with efficient yield in the submerged fermentation process. Moreover, the PCR product confirms the presence of the amylase-coding gene in the three selected isolates. The gene coding for this enzyme can also be cloned to obtain recombinant thermoalkaliphilic enzymes.

Determination of the period of bacterial growth and amylase productivity are important to optimize the time of product recovery. The result obtained in this study indicated that there is appreciable high amylase production from the three isolates under optimized conditions. Crude amylases showed stability at a wide range of temperatures (55–80°C), pH (5–9), and NaCl concentration (0–3 M). This study implies that enzymes of these isolates are thermostable and halotolerant. Amylase isolated from such bacteria in this study has huge potential for starch liquefaction and detergent industries as it has considerable temperature and pH stability. The isolates that were selected in the current study are only three; increasing the number of isolates could also increase the chance of obtaining bacteria with interesting features. To determine the maximum hydrolyzing potential of the enzymes, further purification of amylase should be done. Further searching of thermophilic enzyme-producing microorganisms needs to be done to obtain important industrial enzymes.

## Figures and Tables

**Table 1:** Diameter of average clear zone ratio formed by selected bacteria and the time taken to decolorize iodine solution.

Code	Diameter of average clear zone ratio (mm)	Time to decolorize iodine solution (s)
M1	3.933 ± 0.115^i^	17.0 ± 1.0^d^
M2	4.73 ± 0.115^j^	12.33 ± 0.577^c^
M3	2.36 ± 0.057^e^	72.67 ± 2.082^g^
M4	1.90 ± 0.173^d^	82.67 ± 2.517^i^
M5	1.23 ± 0.057^bc^	97.67 ± 1.528^k^
M6	3.03 ± 0.057^g^	33.67 ± 1.528^f^
M7	3.36 ± 0.1527^h^	30.0 ± 1.0^e^
M8	4.96 ± 0.057^k^	9.67 ± 0.577^b^
M9	2.66 ± 0.152^f^	70.67 ± 1.15^g^
M10	2.40 ± 0.10^e^	75.0 ± 1.0^h^
M11	1.90 ± 0.10^d^	71.67 ± 1.52^g^
M12	1.10 ± 0.10^ab^	110.33 ± 1.528^l^
M13	5.96 ± 0.057^l^	6.67 ± 0.577^a^
M14	1.03 ± 0.057^a^	98.00 ± 1.00^k^
M15	1.366 ± 0.152^c^	91.67 ± 1.528^j^

Mean values within the column followed by the same letter are not significantly different (*P* ≥ 0.05).

**Table 2:** Morphological characterization of bacterial isolates.

Parameters	Bacterial isolates		
Macroscopic and microscopic characterization	M_2_	M_8_	M_13_
Cell shape	Long, rod	Long, rod	Short, rod
Cell arrangement	Chain	Chain	Chain
Colonial pigmentation	White	Creamy	Creamy
Gram staining	+	+	+
Spore staining	+	+	+
Motility test	+	+	+

Key: + = positive; − = negative.

**Table 3:** Biochemical characterization of bacterial isolates.

Parameters	Bacterial isolates		
Biochemical characterization	M2	M8	M13
Catalase test	+	+	+
Anaerobic growth	+	+	+
Oxidase test	+	−	+
Citrate utilization	+	−	+
Urea hydrolysis	+	+	+
Casein hydrolysis	+	+	+
Triple sugar iron test (TSI)			
Carbohydrate fermentation	Only glucose fermentation	Both glucose and lactose fermentation	Only glucose fermentation
TSI (butt)	Yellow	Yellow	Yellow
TSI (slant)	Red	Yellow	Red
TSI (H_2_S) production	+	+	+
TSI (gas) production	+	−	+

Key: + = positive; − = negative.

**Table 4:** The effect of metal divalent cations on crude amylase from the three isolates. Each value represents the mean of three independent assays.

Metal ion	Concentration (mM)	Residual activity (%)		
		M_13_	M_8_	M_2_
Ca	1	91.1	91.6	90.4
	5	93.3	106.3	110.2
	10	108.5	88.4	86.7

Mg	1	85.4	84.5	83.6
	5	79.3	81.5	80.7
	10	76.9	78.3	77.6

Zn	1	48.3	50.2	48.5
	5	19.5	20.7	24.6
	10	17.8	18.4	20.7

None		100	100	100

## Data Availability

The datasets used to support the ﬁndings of this study are available from the corresponding author upon reasonable request.
